# The effects of corticosteroids on COPD lung macrophages: a pooled analysis

**DOI:** 10.1186/s12931-015-0260-0

**Published:** 2015-08-20

**Authors:** Andrew Higham, George Booth, Simon Lea, Thomas Southworth, Jonathan Plumb, Dave Singh

**Affiliations:** Centre for Respiratory Medicine and Allergy, Institute of Inflammation and Repair, Manchester Academic Health Science Centre, The University of Manchester and University Hospital of South Manchester, NHS Foundation Trust, Manchester, UK

**Keywords:** COPD, Corticosteroids, Alveolar macrophages, Inflammation, Cytokines, CXCL8

## Abstract

**Background:**

There is large variation in the therapeutic response to inhaled corticosteroids (ICS) in COPD patients. We present a pooled analysis of our previous studies investigating the effects of corticosteroids on lung macrophages, in order to robustly determine whether corticosteroid sensitivity in COPD cells is reduced compared to controls, and also to evaluate the degree of between individual variation in drug response.

**Methods:**

Data from 20 never smokers (NS), 27 smokers (S) and 45 COPD patients was used. Lung macropahges had been stimulated with lipopolysaccharide (LPS), with or without the corticosteroid dexamethasone, and tumour necrosis factor (TNF)-α, interleukin (IL)-6 and chemokine C-X-C motif ligand (CXCL) 8 production was measured.

**Results:**

There was no difference in the anti-inflammatory effects of corticosteroids when comparing group mean data of COPD patients versus controls. The inhibition of TNF-α and IL-6 was greater than CXCL8. The effects of corticosteroids varied considerably between subjects, particularly at lower corticosteroid concentrations.

**Conclusions:**

We confirm that overall corticosteroid sensitivity in COPD lung macrophages is not reduced compared to controls. The varied effect of corticosteroids between subjects suggests that some individuals have an inherently poor corticosteroid response. The limited suppression of lung macrophage derived CXCL8 may promote neutrophilic inflammation in COPD.

**Electronic supplementary material:**

The online version of this article (doi:10.1186/s12931-015-0260-0) contains supplementary material, which is available to authorized users.

## Introduction

Chronic obstructive pulmonary disease (COPD) is characterised by an abnormal immune response to inhaled noxious particles, most commonly tobacco smoke [1]. Inflammatory cell numbers including macrophages, neutrophils and lymphocytes are increased in the lungs of COPD patients [2]. These cells secrete a range of inflammatory mediators including cytokines, chemokines, proteases and oxidants that contribute to the pathophysiology of COPD [3, 4].

Inhaled corticosteroids (ICS) are anti-inflammatory drugs that are commonly used to treat COPD. Corticosteroids bind to and activate the glucocorticoid receptor (GR) [5], resulting in GR nuclear translocation and the subsequent transrepression of inflammatory gene transcription by inhibition of the activity of transcription factors such as nuclear factor kappa-light-chain-enhancer of activated B cells (NF-κB) [6].

Initial clinical studies of ICS in COPD showed no long term benefit of these drugs in unselected patients [7–9]. However, further clinical trials showed that ICS are effective in the subgroup of COPD patients with a history of exacerbations, and their efficacy is maximised when administered with a long acting beta agonist (LABA) [10, 11]. Furthermore, there is also evidence that COPD patients with sputum eosinophilia have a greater lung function response to corticosteroids [12, 13]. There are clinical concerns about the long term safety of ICS, as these drugs cause side effects such as osteoporosis, skin thinning and pneumonia [14]. The therapeutic index of ICS can be optimised by using these drugs in discrete subgroups of patients most likely to show a beneficial clinical response.

It has been reported that corticosteroids have reduced effects on lung macrophage cytokine production from COPD patients compared to controls [15, 16]. We have failed to reproduce this observation in seven different studies [17–23]. The reason for the contrasting results between studies is unclear. The sample sizes of these previous studies from ourselves and others have often been limited, perhaps causing false positive or false negative results. However, we have consistently shown that the effects of corticosteroids vary between cytokines, with CXCL8 production from both COPD and control macrophages showing reduced corticosteroid sensitivity compared to other cytokines.

This paper presents a pooled analysis of our previous results concerning the effects of corticosteroids on cytokine production from COPD and control lung macrophages, in order to overcome potential issues with limited sample sizes. There were two main objectives; (1) to compare the group average results from COPD patients and controls and (2) to determine the degree of between subject heterogeneity in the *in-vitro* response to corticosteroids in COPD patients. In clinical practice, it is known that the therapeutic response to corticosteroids varies between patients, and we have studied whether the same heterogeneity exists when studying *in-vitro* responses.

## Methods

### Study subjects

We have published seven studies that have investigated the corticosteroid sensitivity of COPD lung macrophages [17–23]. Five of these studies used the same cell culture methodology, and studied full drug concentration response curves; data from these five studies were used in this analysis [18–21, 23]. The other two studies were not included as one study used a different cell culture incubation time [17] while the other used a single corticosteroid concentration [22]. The results from 20 never smokers (NS), 27 smokers (S) and 45 COPD patients who participated in previously published studies were used; the demography of these participants is shown in Table 1. Cells from the same participants had occasionally been cultured on two occasions, the first occasion as fresh cells and the second occasion after freeze—thaw. The results were used in two papers; this was most frequent in NS (*n* = 8). The main analysis presented in this paper uses results from fresh cells only.

**Table 1:** Demographics of the study population

	Resection Macrophages			Bronchoscopy Macrophages		
	NS	S	COPD	NS	S	COPD
n	14	21	35	6	6	10
GOLD I	NA	NA	6	NA	NA	4
GOLD II	NA	NA	22	NA	NA	5
GOLD III	NA	NA	7	NA	NA	1
Age	59.6 (15.1)	69.1 (7.3)	68.0 (7.3)	36.0 (8.9)	43.8 (6.4)	62.1 (8.4)
Sex (M/F)	4/10	9/12	27/8	1/5	3/3	5/5
FEV_1_ (L)	2.2 (0.7)	2.3 (0.8)	1.9 (0.8)	3.0 (0.8)	3.7 (0.9)	2.0 (0.6)
FEV_1_ % Predicted	99.4 (19.0)	94.9 (16.6)	63.6 (18.6)	101.5 (13.1)	107.0 (9.0)	74.4 (21.8)
FVC (L)	3.0 (0.9)	3.0 (0.9)	3.2 (0.7)	3.7 (1.2)	4.8 (1.3)	3.3 (1.1)
FEV_1_/FVC Ratio (%)	76.3 (6.5)	75.2 (4.3)	58.4 (10.5)	81.0 (6.6)	76.7 (5.2)	56.2 (9.9)
Pack Year History	0	43.2 (23.3)	54.9 (26.1)	0	15.9 (8.6)	47.1 (30.2)
Current/ex-smoker	0	10/11	24/11	0	6	7/3
ICS Users	0	0	13	0	0	7

Data shown are mean (sd)

*NS* never smokers, *S* smokers, *FEV1* forced expiratory volume in 1 s, *FVC* forced vital capacity, *ICS* inhaled corticosteroid

COPD was diagnosed based on ≥10 pack years smoking history, typical symptoms and airflow obstruction. COPD patients were mainly GOLD stage II with moderate airflow obstruction, although GOLD I and III patients were also included. The majority of COPD patients were current smokers. Southworth et al. Plumb et al. Lea et al. and Higham et al. [23] used lung macrophages isolated from resected lung tissue from patients undergoing surgical resection for lung cancer, whereas Armstrong et al. [18] used lung macrophages isolated from bronchoscopic sampling (bronchoalveolar lavage [BAL]) from research volunteers who did not have lung cancer. All previous studies were approved by the local research ethics committee, and the participants had provided written informed consent.

### Lung macrophage culture

Lung macrophages were isolated from resected lung tissue and BAL as previously described, and then cultured using the same cell culture methodology [18, 20]. Additional file 1 outlines the method used for cells which underwent freeze-thaw. All the studies adhered macrophages over night before pre-incubating with dexamethasone for 1–2 h, prior to stimulation with lipopolysaccharide (LPS 1 μg/ml, Escherichia Coli B6-026; Sigma-Aldrich) for 24 h. We have previously shown that this is a submaximal LPS concentration [17]. Supernatants were removed and analysed for TNF-α, IL-6 and CXCL8 by enzyme linked immunosorbant assay (ELISA, R & D Systems, Abbingdon, UK); the same immunoassays were used in each study.

### Data analysis

All statistical analysis was performed using GraphPad InStat software (GraphPad Software Inc., La Jolla, California, USA). Normality of data was assessed by the Kolmogorov-Smirnoff test. Mean dexamethasone inhibition of TNF-α, IL-6 and CXCL8 were parametric and therefore compared using a one-way ANOVA. One set of results taken from fresh macrophages was included per subject for the main analysis. The reproducibility of the results was analysed by plotting concentration response curves that had been repeated using fresh lung macrophages and those which had undergone freeze-thaw (NS; *N* = 8). When calculating percentage inhibition, 100 % inhibition was complete inhibition of LPS stimulated release; baseline levels were not subtracted from these values. Univariate correlations were performed using the Spearman rank test. IC_50_ values for dexamethasone (the concentration required to cause 50 % inhibition) were calculated using sigmoidal curve fitting of group mean data.

## Results

### Group mean data

**Fig. 1:**
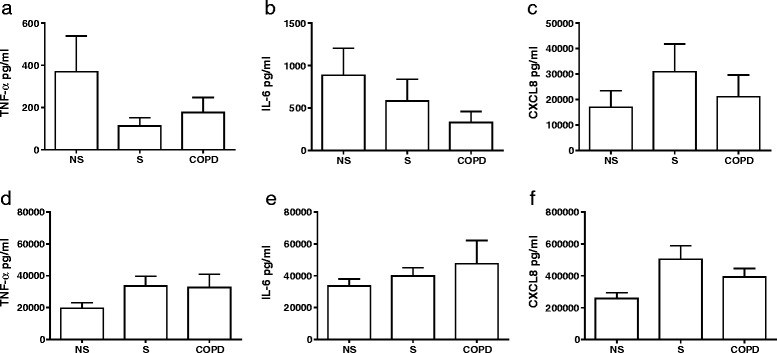
Baseline and LPS stimulated cytokine release. The supernatants from the alveolar macrophages of NS, S and COPD patients were untreated (**a**-**c**) or stimulated with LPS (**d**-**f**) (1 μg/ml) for 24 h and analysed for TNF-α (**a** and **d**), IL-6 (**b** and **e**) and CXCL8 (**c** and **f**) release. Data shown are mean ± SEM

Combining the data using fresh cells from our previous studies, unstimulated and LPS stimulated cytokine levels are shown in Fig. 1. Unstimulated cytokine levels were similar between groups. LPS stimulated cytokine production was also similar between groups.

**Fig. 2:**
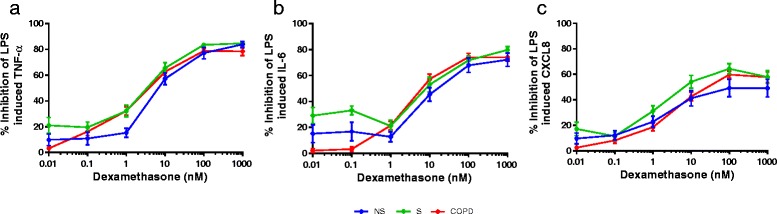
Group mean dexamethasone concentration response curves. The group mean data for dexamethasone inhibition of TNF-α (**a**), IL-6 (**b**) and CXCL8 (**c**) are shown for never smokers (NS; blue plot), smokers (S; green plot) and COPD patients (red plot). Data shown are mean ± SEM

**Fig. 3:**
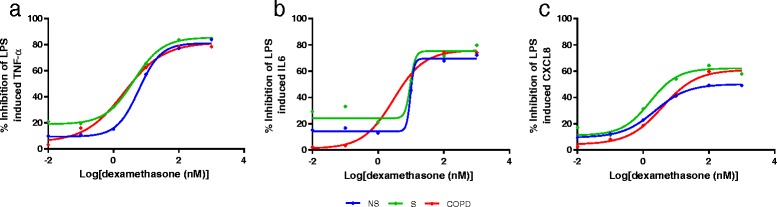
Sigmoidal curve fitting of group mean data. The group mean data for dexamethasone inhibition of TNF-α (**a**), IL-6 (**b**) and CXCL8 (**c**) are shown for never smokers (NS; blue plot), smokers (S; green plot) and COPD patients (red plot)

We constructed dexamethasone concentration response curves (Fig. 2). The effect of dexamethasone was not significantly different between subject groups (*p* > 0.05 for all cytokines at each concentration). We evaluated corticosteroid inhibition further by fitting sigmoidal concentration response curves to the data (Fig. 3). The TNF-α and IL-6 IC_50_ values were very similar in the different subject groups, being in the range 3–10 nM approximately (Table 2). Corticosteroid inhibition of CXCL8 in NS failed to reach 50 %, so we could not calculate the IC_50_. IC_50_ values for CXCL8 inhibition in S and COPD patients were similar (5 nM and 21 nM respectively). In COPD patients, corticosteroid effects were similar in current and ex-smokers, patients taking ICS compared to patients not taking ICS, and males compared to females (Additional file 2).

**Table 2:** IC_50_ values calculated from sigmoidal curve fitting of dexamethasone inhibition

	TNF-α			IL-6			CXCL8		
	NS	S	COPD	NS	S	COPD	NS	S	COPD
IC_50_ (nM)	7.3	3.7	3.4	10.4	9.6	6.2	N/C	5.0	21.0

Data is presented from inhibition of TNF-α, IL-6 and CXCL8 from never smokers (NS), smokers (S) and COPD patients

*N/C* not calculated

The effects of dexamethasone varied between cytokines; the mean inhibition observed at the top of the concentration response curve for TNF-α and IL-6 was up to 80 %, while for CXCL8 this was below 60 %. In all subject groups, the effect of dexamethasone concentrations at the top of the concentration response curve (e.g. 1000 nM) on TNF-α and IL-6 production was significantly greater than the effect on CXCL8 production (*p* < 0.01 for all comparisons in all subject groups).

There were significant correlations (*p* < 0.001 for all analyses) for the corticosteroid inhibition of different cytokines in all subject groups (Additional file 3). The r values for the correlations between cytokines ranged from 0.54 to 0.9, indicating associations varying from moderate to strong.

### Individual data

**Fig. 4:**
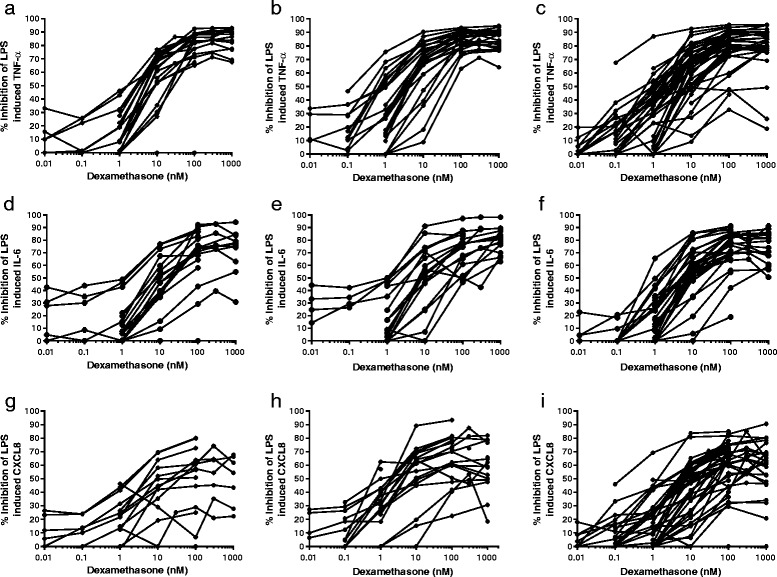
Individual dexamethasone concentration response curves. The individual concentration response curves for dexamethasone inhibition of TNF-α (**a**, **b** and **c**), IL-6 (**d**, **e**, and **f**) and CXCL8 (**g**, **h** and **i**) are shown for never smokers (NS; **a**, **d** and **g**), smokers (S; **b**, **e**, and **h**) and COPD patients (**c**, **f** and **i**)

The individual dexamethasone concentration response curves for TNF-α, IL-6 and CXCL8 using fresh cells only are shown in Fig. 4. Maximal efficacy was achieved at dexamethasone 100 nM - 1000 nM for the majority of subjects. The suppression of TNF-α at these concentrations showed relatively little between subject variation in NS and S, with >60 % inhibition being achieved in most subjects. There was a slight increase in variation for TNF-α with COPD patients at 100 nM - 1000 nM, as 3 patients had <60 % inhibition. However, at concentrations <100 nM there were large variations between COPD patients in the magnitude of TNF-α suppression.

For IL-6 and CXCL8, there was a high degree of variation between patients in the magnitude of inhibition at all dexamethasone concentrations. This was particularly apparent at lower concentrations e.g. at dexamethasone 10 nM the inhibitory effect varied from 0 % to over approximately 80 %, with this pattern observed in both COPD patients and controls.

The percentage of subjects who failed to reach >50 % inhibition by dexamethasone (at any concentration) of TNF-α was less than 10 % (Additional file 4). Similar data were observed for IL-6, with less than 10 % of subjects failing to reach 50 % inhibition by dexamethasone. In contrast, the percentage of subjects who failed to reach >50 % inhibition of CXCL8 was greater; 35 % in NS, 21 % in S and 18 % in COPD patients.

Additional file 5 shows that samples isolated from BAL showed similar results to those from lung resections. There were 8 NS whose cells had been used on two occasions. On the first occasion the cells were used fresh, and on the second occasion the cells had undergone freeze-thaw. Additional file 6 shows that the results of these repeated experiments were very similar.

## Discussion

This pooled analysis confirms that the anti-inflammatory effects of corticosteroids on LPS stimulated lung macrophages does not differ between COPD patients and controls when analysing group mean data. We were also interested to understand the between subject heterogeneity of corticosteroid effect on lung macrophages. The individual concentration response curves showed marked between subject variation, particularly at the lower corticosteroid concentrations. Our results suggest that the overall group mean sensitivity of lung macrophages to corticosteroid treatment is similar in COPD patients and controls, but a proportion of individuals within each group, including COPD patients, display poor *in-vitro* sensitivity to corticosteroids. Perhaps the large between subject variation observed here for the *in-vitro* effects of corticosteroids is relevant to the variable clinical effects of ICS in COPD patients.

The relatively large sample size used for this pooled analysis reduces the chance of false positive or negative results for the group mean data, and supports the concept that the effects of corticosteroids on COPD and control lung macrophages are similar. The studies in this pooled analysis all used identical cell culture methodology, which is important as different methodology can alter the effects of corticosteroid sensitivity experiments through mechanisms such as activation of p38 mitogen activated protein kinase (MAPK) [23]. This pooled analysis also confirms that some cytokines, such as TNF-α and IL-6 show a greater sensitivity to corticosteroid suppression compared to others such as CXCL8. This is probably highly relevant to the clinical effectiveness of corticosteroids in COPD patients, as CXCL8 is present in high concentrations in the lungs of COPD patients [3, 24] and is a key neutrophil chemoattractant [25]. Indeed, we have previously shown that supernatants from stimulated COPD lung macrophages cause neutrophil chemotaxis that is CXCL8 dependent, and that treatment of the lung macrophages with corticosteroids has little effect on chemotaxis [26].

Although we constructed full corticosteroid concentration response curves, it could be argued that the higher concentrations that we studied are clinically less relevant as low non-molar concentrations of ICS are achieved within the lungs [27]; up to 0.6 ng per gram of lung tissue of ICS has previously been observed, which is equivalent to 1.35 nM of drug. We report that the effects of dexamethasone at concentrations from 0.1 to 10 nM is extremely heterogeneous with a high degree of between subject variation, whereas corticosteroid effects at higher concentrations were more homogeneous, particularly for TNF-α. However, the heterogeneous responses at the lower concentrations are more likely to reflect the activity observed in the lungs in real life. At these therapeutically achievable concentrations, our results suggest there is a subgroup of individuals who display relative corticosteroid insensitivity.

The IC_50_ values for TNF-α and IL-6 were similar across the subject groups demonstrating generally similar corticosteroid sensitivities for these cytokines. However, due to the limited effects of corticosteroids on CXCL8 release, we were unable to calculate an IC_50_ value for NS, which is a limitation of this analysis. Furthermore, the group IC_50_ values are not reflective of the wide degree of between individual variation (see Fig. 4) which shows a subgroup of patients with poor corticosteroid sensitivity, particularly at the lower drug concentrations.

The individual concentration response curves identified patients who never reached over 50 % inhibition (up to 35 % of patients for CXCL8). Although this is an arbitrary cut-off point, this observation still demonstrates that in some individuals there is a very limited effect of corticosteroids on lung macrophages, while in other individuals the effect is over 80 % inhibition of cytokine production. Ratcliffe and Dougall reported similar findings with COPD lung macrophages; there was a subset of patients with a reduced response to corticosteroids [28]. Similarly, corticosteroids have heterogeneous effects on alveolar macrophage cytokine production in patients with asthma [29].

There were significant associations between cytokines in the magnitude of corticosteroid inhibition; these relationships were evident in all subject groups. This suggests that low (or high) corticosteroid sensitivity on one cytokine in an individual is associated with a similar effect on other cytokines on an individual basis.

There are various possible mechanisms that could be responsible for the variation in corticosteroid response between individuals. For example, GR expression determines the response to corticosteroids [30]. We have previously shown that the levels of phosphorylated GR in lung macrophages do not differ between COPD patients and controls on a group mean basis [20], supporting the concept that there is no difference in corticosteroid sensitivity between subject groups. However, GR activity may be determined by the relative expression of GR isoforms; whereas GRα is important for mediating the anti-inflammatory actions of glucocorticoids, GRβ has been shown to inhibit the activity of GRα [31]. The levels of GRβ protein have been shown to be increased in BAL macrophages of steroid insensitive asthmatics compared to steroid sensitive asthmatics and this corresponds with reduced nuclear translocation of GRα [32]. More recently it has been shown that the levels of GRβ mRNA are increased in the peripheral blood neutrophils of COPD patients compared to healthy subjects [33].

Activation of the p38 MAPK pathway promotes inflammation by increasing inflammatory gene expression, stabilising mRNAs and enhancing protein translation [34]. Activation of this pathway is corticosteroid insensitive in alveolar macrophages [18], and it has also been shown that the administration of prednisolone to COPD patients does not suppress p38 MAPK activation in whole blood cultured *ex vivo* [35]. In the current analysis, it is possible that inflammatory gene expression in those patients with reduced corticosteroid sensitivity is associated with increased p38 MAPK activity. This may be due to increased phosphorylation of GR at serine 226 by p38 MAPK, which results in reduced nuclear translocation and failure of GR to inhibit gene transcription [36].

A recent study has shown that COPD lung macrophages with different densities demonstrate varied corticosteroid sensitivity [37]. LPS stimulated TNF-α and CXCL8 secretion from COPD macrophages with a lower density were less sensitive to corticosteroid inhibition compared to controls. However, this difference diminished with increasing macrophage density. The current analysis used results from unfractionated macrophages, so we were unable to define the characteristics of cells from individuals with less corticosteroid sensitivity; individuals with a higher proportion of corticosteroid insensitive macrophages within a mixed macrophage population may have a reduced corticosteroid response overall. Furthermore, it is possible that differences in the proportions of macrophage subpopulations between groups may impact the overall group mean corticosteroid sensitivities observed.

Lung tissue as far distal to the tumour as possible from individuals with lung cancer were used, but we cannot rule out the possibility that cancer affects macrophage phenotype. We have controlled for this to some extent by the inclusion of macrophages isolated from BAL from the lungs of subjects who did not suffer from lung cancer. Additional file 5 shows that the corticosteroid response is similar when comparing macrophages isolated from lung tissue compared to BAL.

The studies included in this analysis used LPS to stimulate macrophages. The airways of COPD patients are often colonised with bacteria, making LPS stimulation a physiologically relevant model to study macrophage pharmacology [37–39]. Alternatively, other stimuli such as cigarette smoke extract and IL-1β have been used as inflammatory stimuli for the purpose of studying corticosteroid sensitivity in macrophages [15]. The conclusions that we draw from our analysis are relevant only to LPS stimulation of macrophages.

As a secondary analysis, we compared the results using fresh lung macrophages with those that had undergone freeze-thaw from the same subject. Corticosteroid effects appeared similar, indicating that using frozen cells to study corticosteroid effects is a valid method.

An important finding of this study is the demonstration that lung macrophages from a subset of COPD patients have a high degree of corticosteroid sensitivity at relatively low concentrations. We could speculate that ICS are more likely to exert a clinical benefit in these patients, but this requires prospective evaluation. Sputum and blood eosinophils have been used as biomarkers of corticosteroid response [12, 40] in order to use these drugs in a manner that would optimise the therapeutic index as part of a personalised medicine regime. It would be interesting to know if there is a relationship between the lung macrophage corticosteroid sensitivity reported here and eosinophil numbers. We did not have the opportunity to study the detailed clinical characteristics, including eosinophil counts, of the COPD patients included in this analysis, as the majority were undergoing surgery for lung cancer.

## Conclusions

In conclusion, this pooled analysis supports previous observations that the overall corticosteroid sensitivity of lung macrophages is not reduced in COPD patients [17–23]. The effects of corticosteroids on CXCL8 secretion were limited. Interestingly, there was a large variation between individuals for corticosteroid effects at lower drug concentrations. These results may be clinically relevant, as poor suppression of macrophage activity may result in excessive CXCL8 production promoting persistent neutrophilic inflammation.

## Additional files

Additional file 1:
**Lung macrophage freeze-thaw method.** (DOCX 13 kb) Additional file 2:
**Mean dexamethasone dose response curves comparing COPD current smokers to ex-smokers, ICS users to those not using ICS, and males to females.** Data from COPD patients was separated into current smokers (purple plot) vs ex-smokers (grey plot) (a, b and c), ICS users (black plot) vs those not using ICS (orange plot) (d, e and f) and male (brown plot) vs female (pink plot) patients (g, h and i). Supernatants were analysed for TNF-α (a, d and g), IL-6 (b, e, and h) and CXCL8 (c, f and i). Data shown are mean ± SEM. (PPTX 684 kb) Additional file 3:
**Correlations of corticosteroid cytokine inhibition.** Correleations were made between TNF-α and IL-6 (a–c), TNF-α and CXCL8 (d–f) and IL-6 and CXCL8 (g–i) release from NS (a, d and g), S (b, e, and h) and COPD patients (c, f and i). Data shown are individual data points where r represents the Spearman Rank coefficient. (PPTX 895 kb) Additional file 4:
**The proportion of subjects who displayed less than 50 % or 60 % dexamethasone inhibition of LPS stimulated cytokine production.** Data presented as % of subjects for TNF-α (NS = 20; S = 27; COPD = 45), IL-6 (NS = 18; S = 19; COPD = 31), and CXCL8 (NS = 14; S = 19; COPD = 40). (DOCX 14 kb) Additional file 5:
**Individual dexamethasone dose response curves of macrophages isolated from resected lung tissue and BAL.** Macrophages were isolated from resected lung tissue (black plots) and BAL (red plots) of never smokers (NS; a, d and g), smokers (S; b, e, and h) and COPD patients (c, f and i). Supernatants were analysed for TNF-α (a, b and c), IL-6 (d, e, and f) and CXCL8 (g, h and i). (PPTX 1404 kb) Additional file 6:
**Reproducibility of dexamethasone concentration response curves.** Lung macrophages from 8 NS were used on two separate occasions – experiment 1 (grey circles) and experiment 2 (black triangles). Dexamethasone concentration response curves are shown for TNF-α. (PPTX 708 kb)
