# Supplementary material for: The effects of corticosteroids on COPD lung macrophages: a pooled analysis
Source: Respir Res. 2015 Aug 20;16(1):98. doi: 10.1186/s12931-015-0260-0 (PMC4545868; doi:10.1186/s12931-015-0260-0)
Supplement: Additional file 1: — Lung macrophage freeze-thaw method. (DOCX 13 kb) [file 12931_2015_260_MOESM1_ESM.docx]

**METHODS**

**Lung Macrophage Freeze-Thaw**

Lung macrophages isolated from resected lung tissue were used fresh, or frozen in a suspension of foetal calf serum (FCS; Invitrogen, Paisley, UK) containing 10% DMSO and stored in liquid nitrogen until required. Upon thawing, the macrophage suspension was added to RPMI-1640 media and centrifuged (400 g for 10 minutes at 4^o^C). Macrophage viability was assessed by trypan blue exclusion. Macrophages were re-suspended in RPMI-1640 media supplemented with 10% v/v FCS, 2 mM L-glutamine (Invitrogen), 100 U/ml penicillin and 100 μg/ml streptomycin (Sigma-Aldrich)’. 0.1 x 10^6^ viable macrophages were seeded per well. The following day non-adherent cells were removed.
