# Supplementary material for: The effects of corticosteroids on COPD lung macrophages: a pooled analysis
Source: Respir Res. 2015 Aug 20;16(1):98. doi: 10.1186/s12931-015-0260-0 (PMC4545868; doi:10.1186/s12931-015-0260-0)
Supplement: Additional file 2: — Mean dexamethasone dose response curves comparing COPD current smokers to ex-smokers, ICS users to those not using ICS, and males to females. Data from COPD patients was separated into current smokers (purple plot) vs ex-smokers (grey plot) (a, b and c), ICS users (black plot) vs those not using ICS (orange plot) (d, e and f) and male (brown plot) vs female (pink plot) patients (g, h and i). Supernatants were analysed for TNF-α (a, d and g), IL-6 (b, e, and h) and CXCL8 (c, f and i). Data shown are mean ± SEM. (PPTX 684 kb) [file 12931_2015_260_MOESM2_ESM.pptx]

## Slide 1
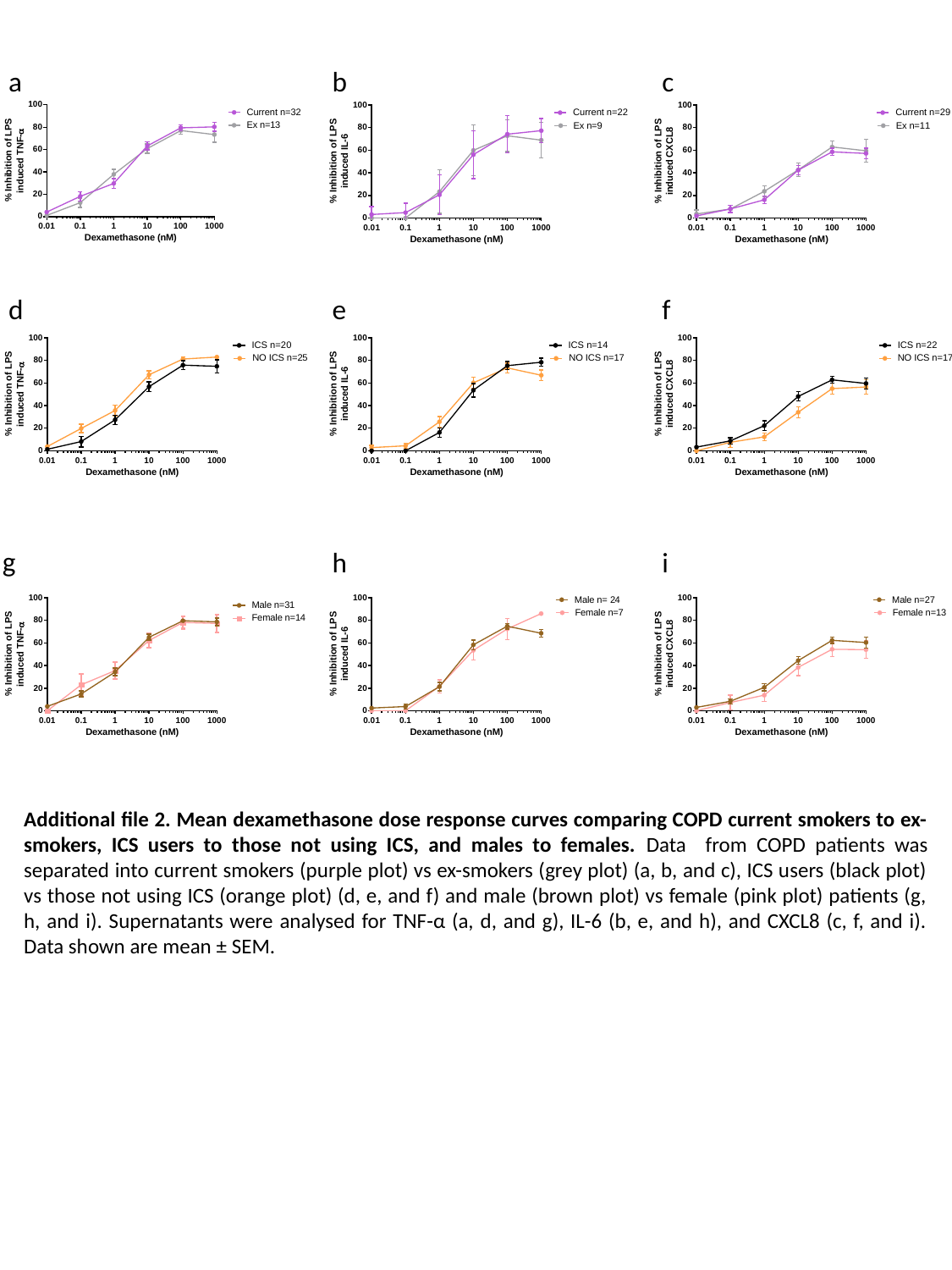

a
b
c
d
e
f
g
h
i
Additional file 2. Mean dexamethasone dose response curves comparing COPD current smokers to ex-smokers, ICS users to those not using ICS, and males to females. Data from COPD patients was separated into current smokers (purple plot) vs ex-smokers (grey plot) (a, b, and c), ICS users (black plot) vs those not using ICS (orange plot) (d, e, and f) and male (brown plot) vs female (pink plot) patients (g, h, and i). Supernatants were analysed for TNF-α (a, d, and g), IL-6 (b, e, and h), and CXCL8 (c, f, and i). Data shown are mean ± SEM.
