# Supplementary material for: The effects of corticosteroids on COPD lung macrophages: a pooled analysis
Source: Respir Res. 2015 Aug 20;16(1):98. doi: 10.1186/s12931-015-0260-0 (PMC4545868; doi:10.1186/s12931-015-0260-0)
Supplement: Additional file 3: — Correlations of corticosteroid cytokine inhibition. Correleations were made between TNF-α and IL-6 (a–c), TNF-α and CXCL8 (d–f) and IL-6 and CXCL8 (g–i) release from NS (a, d and g), S (b, e, and h) and COPD patients (c, f and i). Data shown are individual data points where r represents the Spearman Rank coefficient. (PPTX 895 kb) [file 12931_2015_260_MOESM3_ESM.pptx]

## Slide 1
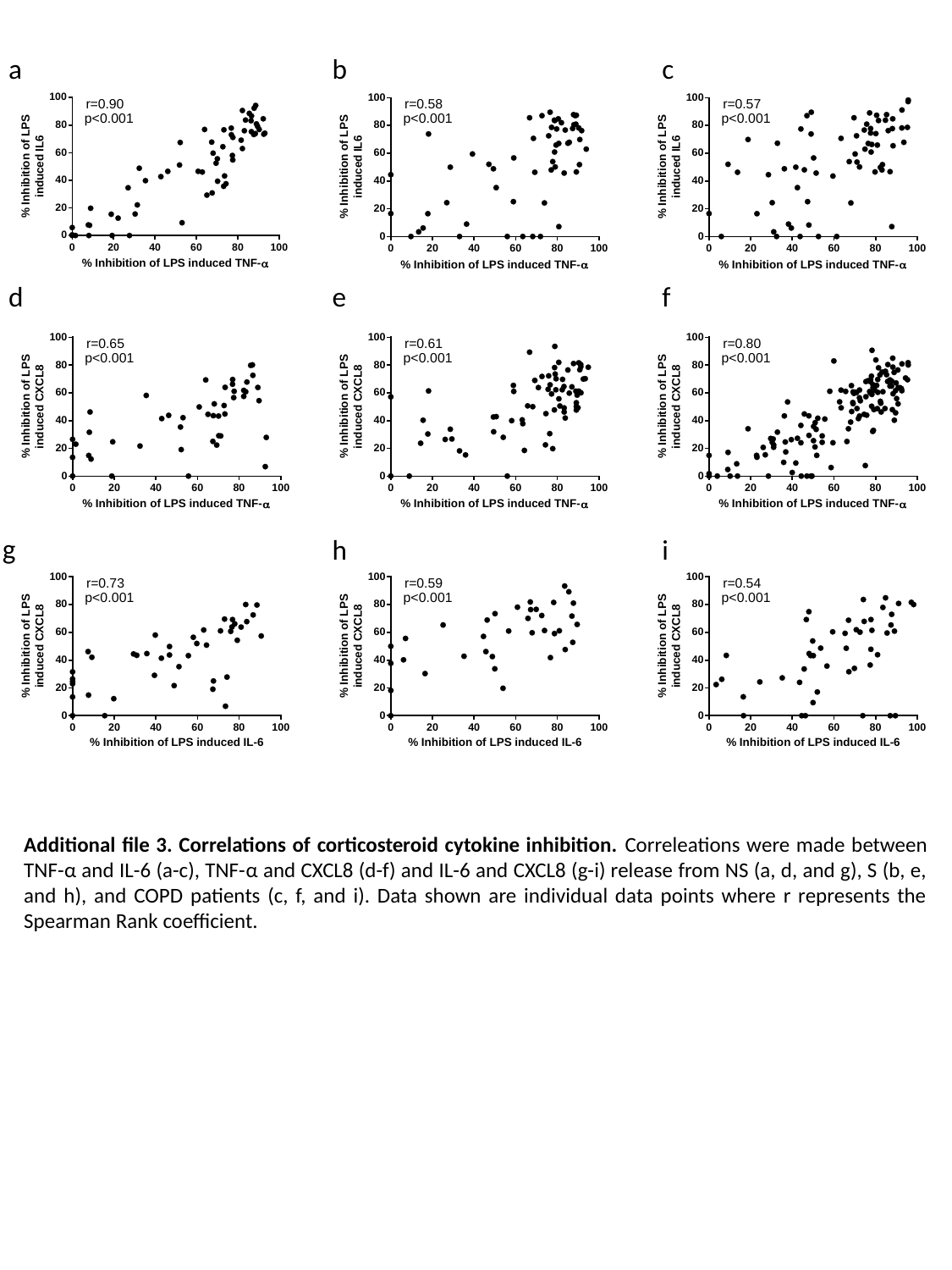

a
b
c
d
e
f
g
h
i
Additional file 3. Correlations of corticosteroid cytokine inhibition. Correleations were made between TNF-α and IL-6 (a-c), TNF-α and CXCL8 (d-f) and IL-6 and CXCL8 (g-i) release from NS (a, d, and g), S (b, e, and h), and COPD patients (c, f, and i). Data shown are individual data points where r represents the Spearman Rank coefficient.
