# Supplementary material for: The effects of corticosteroids on COPD lung macrophages: a pooled analysis
Source: Respir Res. 2015 Aug 20;16(1):98. doi: 10.1186/s12931-015-0260-0 (PMC4545868; doi:10.1186/s12931-015-0260-0)
Supplement: Additional file 4: — The proportion of subjects who displayed less than 50 % or 60 % dexamethasone inhibition of LPS stimulated cytokine production. Data presented as % of subjects for TNF-α (NS = 20; S = 27; COPD = 45), IL-6 (NS = 18; S = 19; COPD = 31), and CXCL8 (NS = 14; S = 19; COPD = 40). (DOCX 14 kb) [file 12931_2015_260_MOESM4_ESM.docx]

|  | **< 50% Inhibition** | | | **< 60% Inhibition** | | |
| --- | --- | --- | --- | --- | --- | --- |
|  | **NS** | **S** | **COPD** | **NS** | **S** | **COPD** |
| **TNF-α** | 5% | 0 | 9% | 5% | 0 | 9% |
| **IL-6** | 11% | 0 | 3% | 22% | 0 | 10% |
| **CXCL8** | 35% | 21% | 18% | 50% | 21% | 23% |

**Additional file 4. The proportion of subjects who displayed less than 50% or 60% dexamethasone inhibition of LPS stimulated TNF-α (NS=20; S=27; COPD=45), IL-6 (NS=18; S=19; COPD=31), and CXCL8 (NS=14; S=19; COPD=40).** Data presented as % of subjects.
