# Supplementary material for: The effects of corticosteroids on COPD lung macrophages: a pooled analysis
Source: Respir Res. 2015 Aug 20;16(1):98. doi: 10.1186/s12931-015-0260-0 (PMC4545868; doi:10.1186/s12931-015-0260-0)
Supplement: Additional file 5: — Individual dexamethasone dose response curves of macrophages isolated from resected lung tissue and BAL. Macrophages were isolated from resected lung tissue (black plots) and BAL (red plots) of never smokers (NS; a, d and g), smokers (S; b, e, and h) and COPD patients (c, f and i). Supernatants were analysed for TNF-α (a, b and c), IL-6 (d, e, and f) and CXCL8 (g, h and i). (PPTX 1404 kb) [file 12931_2015_260_MOESM5_ESM.pptx]

## Slide 1
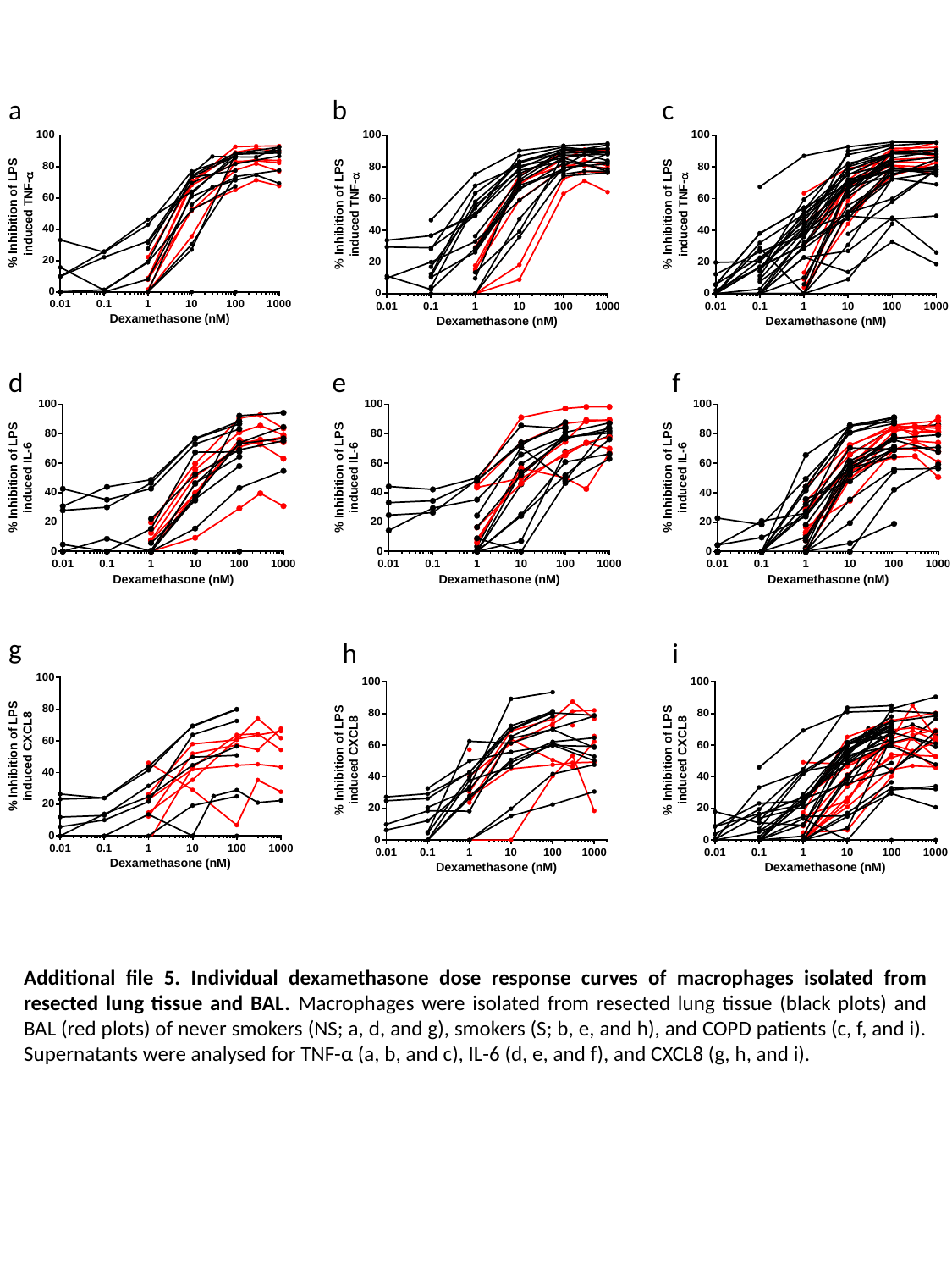

a
b
c
d
e
f
g
h
i
Additional file 5. Individual dexamethasone dose response curves of macrophages isolated from resected lung tissue and BAL. Macrophages were isolated from resected lung tissue (black plots) and BAL (red plots) of never smokers (NS; a, d, and g), smokers (S; b, e, and h), and COPD patients (c, f, and i). Supernatants were analysed for TNF-α (a, b, and c), IL-6 (d, e, and f), and CXCL8 (g, h, and i).
