# Supplementary material for: The effects of corticosteroids on COPD lung macrophages: a pooled analysis
Source: Respir Res. 2015 Aug 20;16(1):98. doi: 10.1186/s12931-015-0260-0 (PMC4545868; doi:10.1186/s12931-015-0260-0)
Supplement: Additional file 6: — Reproducibility of dexamethasone concentration response curves. Lung macrophages from 8 NS were used on two separate occasions – experiment 1 (grey circles) and experiment 2 (black triangles). Dexamethasone concentration response curves are shown for TNF-α. (PPTX 708 kb) [file 12931_2015_260_MOESM6_ESM.pptx]

## Slide 1
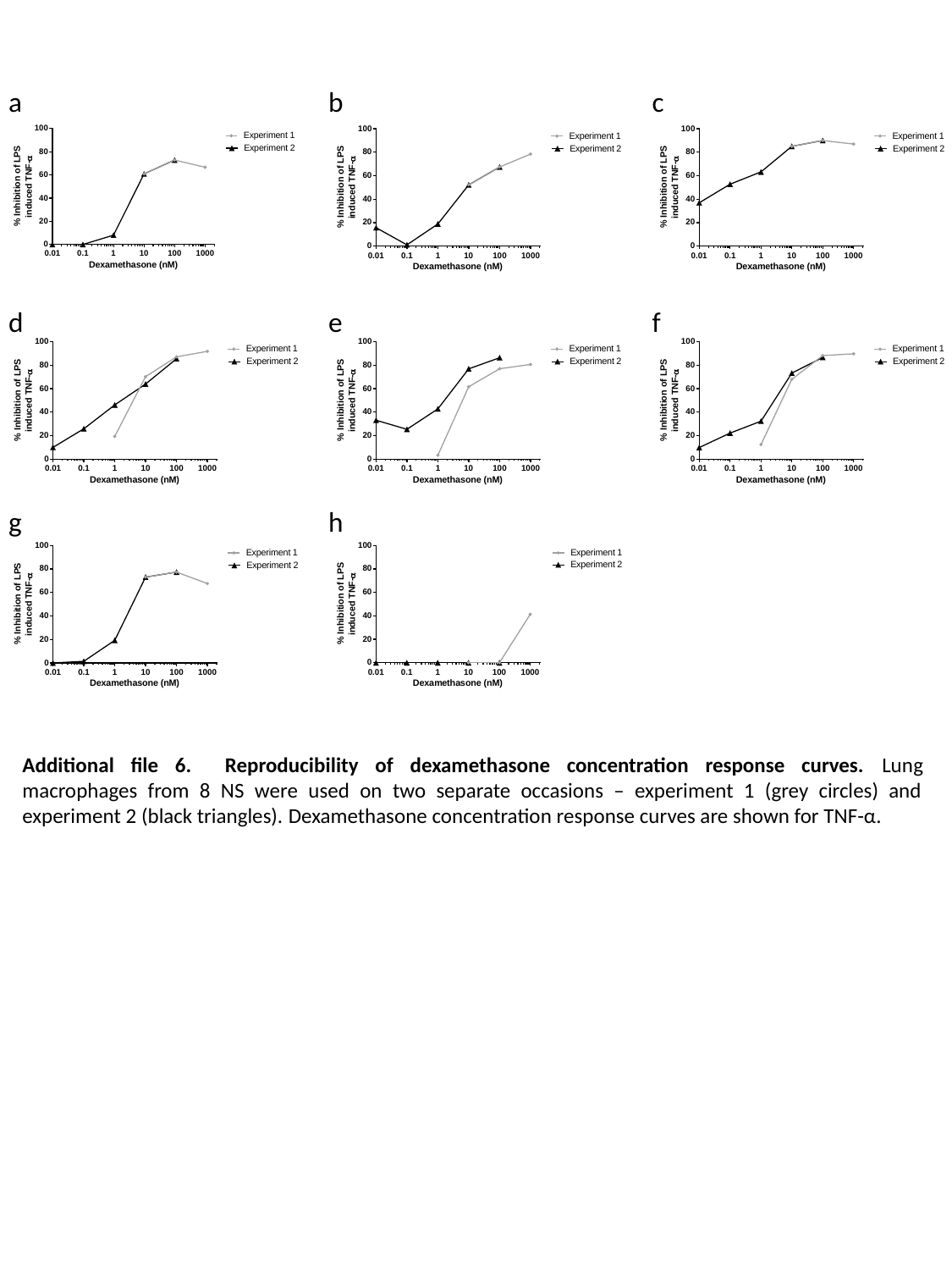

a
b
c
d
e
f
g
h
Additional file 6. Reproducibility of dexamethasone concentration response curves. Lung macrophages from 8 NS were used on two separate occasions – experiment 1 (grey circles) and experiment 2 (black triangles). Dexamethasone concentration response curves are shown for TNF-α.
